# Household level spatio-temporal analysis of *Plasmodium falciparum* and *Plasmodium vivax* malaria in Ethiopia

**DOI:** 10.1186/s13071-017-2124-6

**Published:** 2017-04-20

**Authors:** Dinberu Seyoum, Delenasaw Yewhalaw, Luc Duchateau, Patrick Brandt, Angel Rosas-Aguirre, Niko Speybroeck

**Affiliations:** 1 Institute of Health and Society (IRSS), Université catholique de Louvain, Brussels, Belgium; 2 Department of Statistics, Natural Science College, Jimma University, Jimma, Ethiopia; 3 Department of Medical Laboratory Sciences and Pathology, College of Health a Sciences, Jimma University, Jimma, Ethiopia; 4 Tropical and Infectious Diseases Research Center, Jimma University, Jimma, Ethiopia; 5 Department of Comparative Physiology and Biometrics, Faculty of Veterinary Medicine, Ghent University, Gent, Belgium; 6 School of Economic, Political and Policy Sciences, The University of Texas, Dallas, USA; 7 Institute of Tropical Medicine “Alexander von Humboldt”, Universidad Peruana Cayetano Heredia, Lima, Peru

**Keywords:** Spatio-temporal analysis, SatScan, *Plasmodium vivax*, *Plasmodium falciparum*, Active case detection, Ethiopia

## Abstract

**Background:**

The global decline of malaria burden and goals for elimination has led to an increased interest in the fine-scale epidemiology of malaria. Micro-geographic heterogeneity of malaria infection could have implications for designing targeted small-area interventions.

**Methods:**

Two-year longitudinal cohort study data were used to explore the spatial and spatio-temporal distribution of malaria episodes in 2040 children aged < 10 years in 16 villages near the Gilgel-Gibe hydropower dam in Southwest Ethiopia. All selected households (HHs) were geo-referenced, and children were followed up through weekly house-to-house visits for two consecutive years to identify febrile episodes of *P. falciparum* and *P. vivax* infections. After confirming the spatial dependence of malaria episodes with Ripley’s K function, SatScan^TM^ was used to identify purely spatial and space-time clusters (hotspots) of annual malaria incidence for 2 years follow-up: year 1 (July 2008-June 2009) and year 2 (July 2009-June 2010).

**Results:**

In total, 685 *P. falciparum* episodes (in 492 HHs) and 385 *P. vivax* episodes (in 290 HHs) were identified, representing respectively incidence rates of 14.6 (95% CI: 13.4–15.6) and 8.2 (95% CI: 7.3–9.1) per 1000 child-months at risk. In year 1, the most likely (128 HHs with 63 episodes, RR = 2.1) and secondary (15 HHs with 12 episodes, RR = 5.31) clusters of *P. vivax* incidence were found respectively in southern and north-western villages; while in year 2, the most likely cluster was located only in north-western villages (85 HHs with 16 episodes, RR = 4.4). Instead, most likely spatial clusters of *P. falciparum* incidence were consistently located in villages south of the dam in both years: year 1 (167 HHs with 81 episodes, RR = 1.8) and year 2 (133 HHs with 67 episodes, RR = 2.2). Space-time clusters in southern villages for *P. vivax* were found in August-November 2008 in year 1 and between November 2009 and February 2010 in year 2; while for *P. falciparum*, they were found in September-November 2008 in year 1 and October-November 2009 in year 2.

**Conclusion:**

Hotspots of *P. falciparum* incidence in children were more stable at the geographical level and over time compared to those of *P. vivax* incidence during the study period.

**Electronic supplementary material:**

The online version of this article (doi:10.1186/s13071-017-2124-6) contains supplementary material, which is available to authorized users.

## Background

Despite a decline in the global malaria burden over the past 15 years, about 3.5 billion people were at risk worldwide in 2015, and millions of them are still not accessing the services they need to prevent and treat malaria k. Of 438,000 registered malaria deaths in 2015, approximately 80% of the deaths were concentrated in just 15 countries, mainly in Africa [1]. According to the Ethiopian Ministry of Health [2], 2,174,707 malaria clinical cases and 662 deaths due to malaria were registered between September 2014 and August 2015 (Ethiopian fiscal year - EFY 2014/2015). Laboratory confirmation of malaria by either light microscopy (LM) or rapid diagnostic tests (RDTs) was performed in 1,867,059 (85.9%) clinical cases, showing a predominance of *Plasmodium falciparum* (63.7%) over *P. vivax* cases (36.3%). Oromia is the second regional state of Ethiopia with the highest malaria incidence, accounting for about 20% (430,969 cases) of total reported clinical cases in the country (430,969 cases), but the first one in terms of malaria mortality, representing about one third (214 deaths) of total malaria-related deaths in Ethiopia [2].

Malaria transmission is mainly seasonal and unstable throughout the country and varies due to differences in altitude, season, and population movement [3, 4]. A good understanding of the local epidemiology and transmission dynamics of malaria infections is key for better targeting the control measures [5–8]. Many factors have been reported to significantly influence malaria transmission in Ethiopia with likely different levels of interaction across space and time [9, 10]. Ecological factors facilitating breeding sites of *Anopheles arabiensis* (i.e. dams, irrigation canals, floods on shorelines, agricultural field puddles, wet lands, man-made pools, and rain pools) [11, 12] or resting places for adult mosquitos (i.e. surrounding vegetation, housing characteristics) are thought to be the main factors for malaria transmission [13]. Conditions that increase exposure to infectious mosquitos’ bites (e.g. agriculture and livestock economic activities) [14], and human behavioural factors that limit the coverage and effectiveness of malaria control interventions (e.g. outdoor sleeping habits, low utilization of long-lasting insecticidal nets, poor treatment seeking behaviours, and low treatment adherence) may also influence the malaria risk [15].

A previous analysis of malaria surveillance data based on passive case detection (PCD) in villages located around the Gilgel-Gibe hydroelectric power dam in Southwest Ethiopia suggests different spatial and temporal variations of malaria episodes for both *P. falciparum* and *P. vivax* [10]. Until now the use of spatial-temporal tools to detect malaria hotspots (i.e. single villages or groups of households within villages with increased risk of malaria transmission) has not been applied to analyse malaria transmission in this area. Capitalising on the availability of two-year longitudinal malaria cohort data, this study explored the spatial and spatio-temporal distribution of *P. falciparum* and *P. vivax* malaria episodes in 2040 children aged < 10 years living in 16 villages around the Gilgel-Gibe hydropower dam. The study was conducted as part of several other studies, intended to assess the impact of the Gilgel-Gibe hydroelectric dam on health and other sectors (environment, agriculture and economy) following its starting operation in 2004 [16].

## Methods

### Study area

**Fig. 1:**
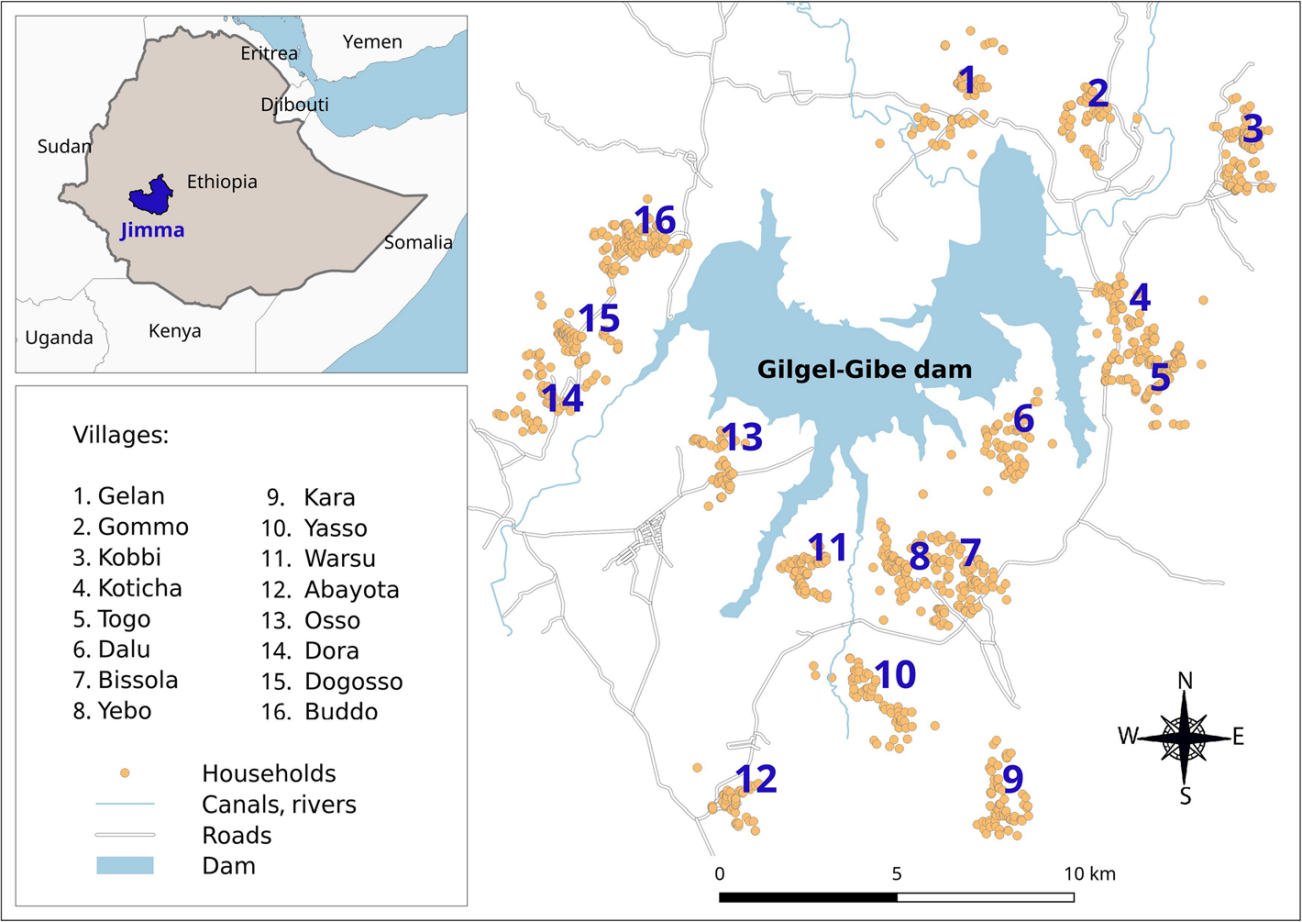
Study villages around the Gilgel-Gibe hydroelectric dam reservoir, Jimma zone, Ethiopia

The study was conducted in Gilgel-Gibe dam area, in Jimma zone (Fig. 1), which is located 260 km south-west of Addis Ababa, in the Oromia region of Ethiopia. The study area lies between latitudes 7°42′50″N and 07°53′50″N and between longitudes 37°11′22″E and 37°20′36″E, at an altitude of 1734–1864 m above sea level. Sixteen villages within a 10 km radius (265–9046 m) from the dam reservoir shore were randomly selected based on similar eco-topography, access to health facilities, and homogeneity with respect to socio-cultural and economic activities [17, 18]. The main socio-economic activities of the households are mixed farming involving the cultivation of staple crops (maize, teff and sorghum), cattle, and small stock. All the households residing in the study villages belong to the Oromo ethnic group, which is one of the largest ethnic groups in Ethiopia [11].

### Study design and population

A longitudinal 2-year malaria cohort study was conducted in children under 10 years old, living in the 16 selected villages around the Gilgel-Gibe hydroelectric power dam. A total of 2040 children aged less than 10 years were enrolled in July 2008 and completed weekly follow-ups until June 2010 [17]. Each child was identified with a unique code, and selected villages and households were geo-referenced using a handheld global positioning system (GPS) device (Garmin’s GPSMAP 60CSx, Garmin International Inc., Olathe, Kansas, USA).

### Follow-up, identification and management of malaria episodes

Active case detection (ACD) through weekly household visits was conducted to identify and register all febrile malaria episodes in the study population during the two-year follow-up period. During the household visits, axillary body temperature was taken, and the caregiver was asked about fever history. If a child had a fever (temperature ≥ 37.5 °C) or reported a history of fever in the past 24 h a finger-prick blood sample was taken for immediate diagnosis by LM in the same site, or at Omo-Nada District Health Center Laboratory. Trained laboratory technicians conducted LM diagnosis. Thick smears were used to confirm the presence or absence of parasites, whereas the thin smear was used to identify the *Plasmodium* species.

All children with malaria confirmed by LM were treated according to the national treatment guidelines [19]. Treatment was administered by the parents and/or caregivers of the children, and consisted of 25 mg/kg of chloroquine (CQ) over three consecutive days for *P. vivax*, and artemether-lumefantrine (AL) for *P. falciparum* twice daily according to the body weight as follows: 5–14 kg, one tablet per dose; 15–24 kg, two tablets per dose; 25–34 kg, three tablets per dose; and adults, four tablets per dose. Treatment adherence was monitored during household visits by asking for the medication packages and the remaining pills. Absent children were followed in the next visits, and their caregivers were asked about the occurrence of symptomatic episodes and the confirmation of malaria at the health facility. In addition, all the health facilities near the study communities were visited monthly to verify their clinical records, checking if any enrolled children had presented a confirmed malaria episode in the past month, not being detected during the weekly visits.

### Data analysis

#### Global spatial clustering

Data were double entered, validated and cleaned in Excel (Microsoft Corp, USA). A univariate Ripley’s K-function was used to assess whether children with malaria episodes tended to be near other children with episodes (determination of the expected number of children with episodes within distance ***h*** of a given child with episodes), while a bivariate K-function explored the spatial independence between children classified into two groups according to specific conditions (determination of the expected number of children with condition 1 within distance ***h*** of children with condition 2) [20].

For each malaria species and year of follow-up (first or second year), a file was created with the coordinates of children’s households. The file was read in R software as a table, and a grid was created using the maximum and minimum values of latitude and longitude coordinates. After creating an object of class “ppp” for the point pattern distribution of children in a polygonal window, the Kest and Lest functions from the package Spatstat were applied to plot both observed and expected K-values over a range of distances [21]. Expected K-values and corresponding 95% confidence envelopes (CEs) were calculated using 999 Monte Carlo simulations to test the null hypothesis (***H***_***o***_). The univariate K-function tested the ***H***_***o***_ that the children with malaria episodes were randomly spatially distributed (complete spatial randomness, CSR), while the bivariate K-function tested two separated ***H***_***o***_: a) the children with and without malaria episodes were independently spatial distributed, and b) the children with malaria episodes, younger than 3 years old and older than 3 years old, were independently spatial distributed.

In the univariate analysis, observed K-values between low and high CE at specific distance ***h*** indicated random distribution of the children with malaria episodes for that distance ***h***, while those larger than the high CE values or those smaller than the low CE values at specific distance ***h*** indicated respectively significant spatial clustering or spatial dispersion of children with malaria episodes for that distance ***h***. In the bivariate analysis, observed difference of K values between the children group 1 and group 2 which were between low and high CE at a specific distance ***h*** indicated spatial independence between the children groups for that distance ***h***. Instead, a difference of K values larger than the high CE values at specific distance ***h*** indicated that the children group 1 tended to be more clustered than children group 2 for that distance ***h***, while a difference smaller than the low CE values indicated that the household group 1 tends to be more dispersed than household group 2. In all analyses, distances ***h*** ranged from 0 to the maximum distance between the two closest children’s households (about 5 km).

#### Local spatial clustering

The QGIS software v.2.16 (QGIS developer team, Open Source Geospatial Foundation) [22] was used to map all households with children aged less than 10 years old in the study area, and the SaTScan software v.9.3 (M Kulldorff and Information Management Services Inc, Boston, USA) [23, 24] was employed to detect spatial and space-time clusters of *P. falciparum* and *P. vivax* malaria episodes using the Bernoulli probability model [25]. The Bernoulli model in SaTScan requires input data as cases and controls. For each week of follow-up, cases were children with species-specific malaria episodes, while controls were children without episodes. Of note, malaria episodes were only counted for the week they were initially identified, and a child with a malaria episode (treated according to national guidelines) was censored for 21 days to prevent double counting of episodes in successive weeks.

The spatial analysis tests the null hypothesis of no clustering of children with malaria episodes. Different windows with varying size, from zero to a maximum radius of less than 15% of the total children, were allowed to move across the study area. This maximum radius was selected to avoid large non-populated areas within the identified malaria clusters by SaTScan. More details about the selection of maximum windows size can be found in Additional files 1 and 2. Each circle was a candidate cluster for which the log likelihood ratio (LLR) and the relative risk (RR) were obtained. The circular window with the highest LLR was defined as the most likely cluster (hotspot) if the *P*-value < 0.05 [24]. Once the hotspot was identified, a re-analysis of the children within that hotspot was conducted to identify whether that hotspot hid a smaller and more homogeneous area with the highest malaria incidence (i.e. a hotspot within hotspot) [5].

The space-time analysis was performed under the null hypothesis that the risk of having malaria episodes was the same in all households and over time, with cylindrical windows having a circular geographic base and height corresponding to the time scale in weeks. The radius of each circular base was allowed to vary in size, to include up to as many as 15% of the total children. Comparably, the height of the cylinder varied in size up to a maximum of 50% of the study period with a time precision of one week. An unlimited number of overlapping cylinders with different dimensions were obtained, each cylinder corresponding to a possible space-time cluster. For each space-time cluster, the LLR was calculated and the most likely cluster defined as the cylinder with the maximum LLR. The statistical significance of the clusters was tested through 999 Monte Carlo simulations (the default value of the software) to achieve strong power, and the null hypothesis was rejected when the resulting p-value was below 0.05.

## Results

Of the total 2040 followed-up children, 981 (48.1%) were females and 1059 (51.9%) males. The mean age at enrollment was 4.9 ± 2.0 years, not varying significantly across villages (*P* > 0.05) (Table 1). Of the total reported 1070 malaria episodes, 685 (363 episodes in year 1 and 322 episodes in year 2) were due to *P. falciparum* in 492 HHs, and 385 (296 episodes in year 1 and 89 episodes in year 2) were due to *P. vivax* in 290 HHs. *P. falciparum* incidence rates were respectively 15.5 and 13.7 episodes/1000 child-months in the first and second year of follow-up (14.6 episodes/1000 child-months thorough the study period), while *P. vivax* incidence rates were respectively 12.6 and 3.8 episodes/1000 child-months in the first and second year of follow-up (8.2 episodes/1000 child-months thorough the study period). Additional files 3 and 4 show videos with the spatio-temporal distribution of species-specific malaria incidence by HH. The visual inspection from first video suggests seasonal spatial distribution of *P. falciparum* incidence, with increased occurrence of *P. falciparum* episodes in households located at south of the dam mainly in the last months of the long rainy season (August and September), as well as, in the first months of the dry season (October and November). Instead, the second video shows a decreasing trend in the occurrence of *P. vivax* episodes along the study period, with a less clear spatial and seasonal pattern.

**Table 1:** Baseline characteristics of enrolled children and incidence of malaria episodes by village

ID	Villages	Total	Age	Female	Number of episodes					
		*N*	Mean ± SD	%	*P. falciparum*			*P. vivax*		
					Year 1	Year 2	Total	Year 1	Year 2	Total
1	Gelan	133	4.9 ± 1.9	51.1	16	7	23	12	1	13
2	Gommo	127	5.3 ± 2.2	44.9	9	8	17	2	2	4
3	Kobbi	130	4.9 ± 1.7	43.8	5	7	12	2	2	4
4	Koticha	133	4.7 ± 1.8	51.1	16	9	25	11	1	12
5	Togo	124	5.2 ± 1.9	47.6	7	6	13	7	0	7
6	Dalu	136	4.5 ± 2.1	58.8	33	31	64	26	6	32
7	Bissola	134	4.8 ± 2.0	47.1	36	42	78	33	12	45
8	Yebo	126	5.3 ± 2.2	46.8	21	25	46	17	0	17
9	Kara	114	4.4 ± 1.9	42.1	38	32	70	27	8	35
10	Yasso	121	5.4 ± 2.1	43.8	26	29	55	30	11	41
11	Warsu	127	4.3 ± 2.1	43.3	27	16	43	27	8	35
12	Abayota	125	5.3 ± 2.1	56.0	34	30	64	20	4	24
13	Osso	129	5.6 ± 1.9	46.5	22	22	44	22	7	29
14	Dora	125	5.6 ± 1.4	42.4	17	11	28	16	3	19
15	Dogosso	127	5.3 ± 1.5	52.8	28	8	36	11	8	19
16	Buddo	129	4.0 ± 2.3	49.6	28	39	67	33	16	49
Total		2040	4.9 ± 2.0	48.1	363	322	685	296	89	385

*Abbreviation*: *SD* standard deviation



**Additional file 3:** Spatio-temporal distribution of households presenting *P. falciparum* episodes. Each yellow point represents an individual household. When a *P. falciparum* episode in a child is found in the household, the point becomes red. (MP4 862 kb)




**Additional file 4:** Spatio-temporal distribution of households presenting *P. vivax* episodes. Each yellow point represents an individual household. When a *P. vivax* episode is found in a child in the household, the point becomes red. (MP4 769 kb)


### Global spatial clustering

**Fig. 2:**
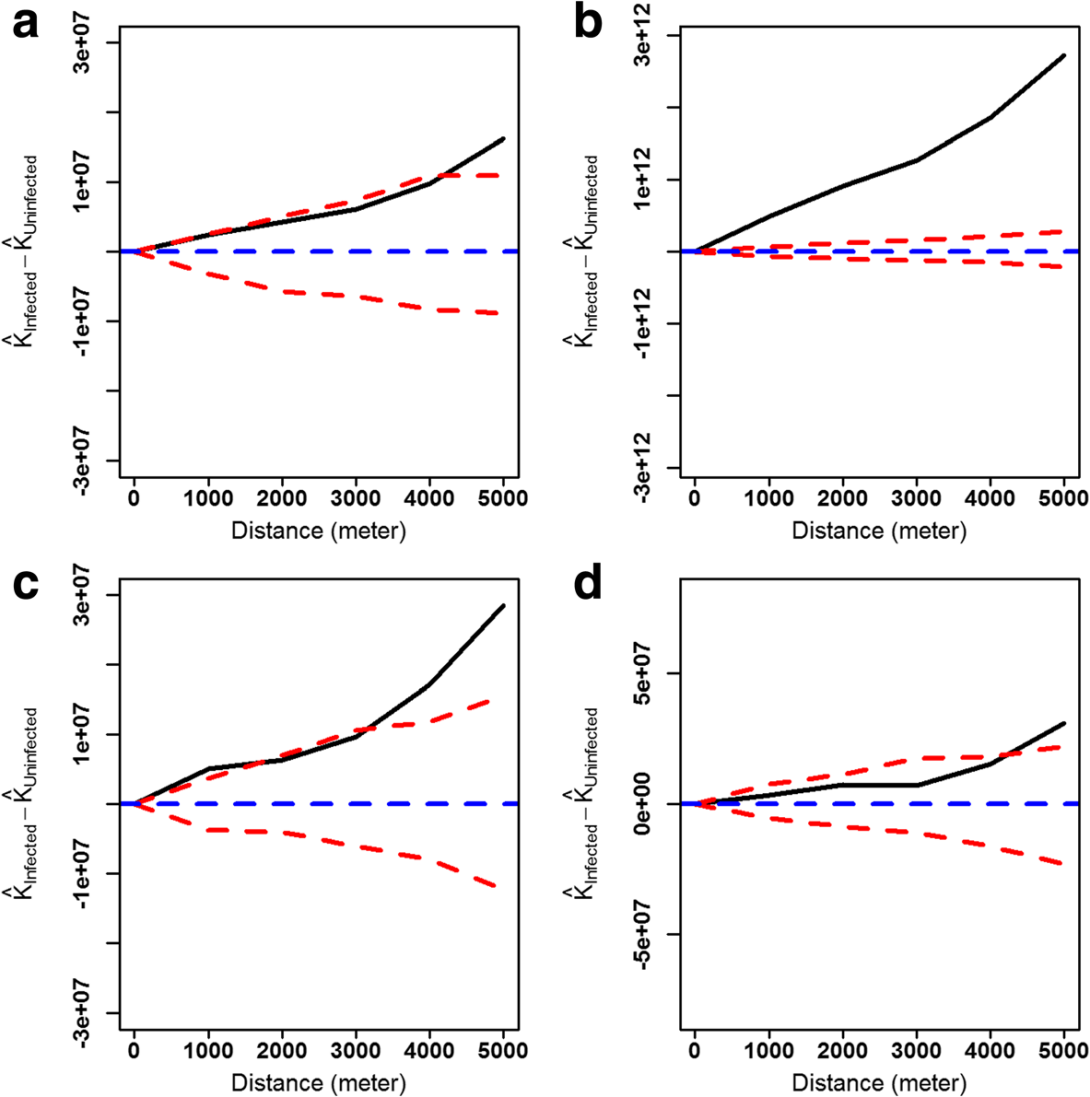
Bivariate Ripley’s K function analysis comparing the spatial distribution of children with and without malaria episodes: **a** for *P. falciparum* incidence in the first year, **b** for *P. falciparum* incidence in the second year, **c** for *P. vivax* incidence in the first year, and **d** for *P. vivax* incidence in the second year. The *blue line* represents the expected difference of K function values (K_infected_-K_noninfected_) between children groups under the null hypothesis of spatial independence, the *solid black line* represents the observed difference K function, *dashed red lines* represents the confidence envelopes for expected K-function values calculated from 999 simulations

**Fig. 3:**
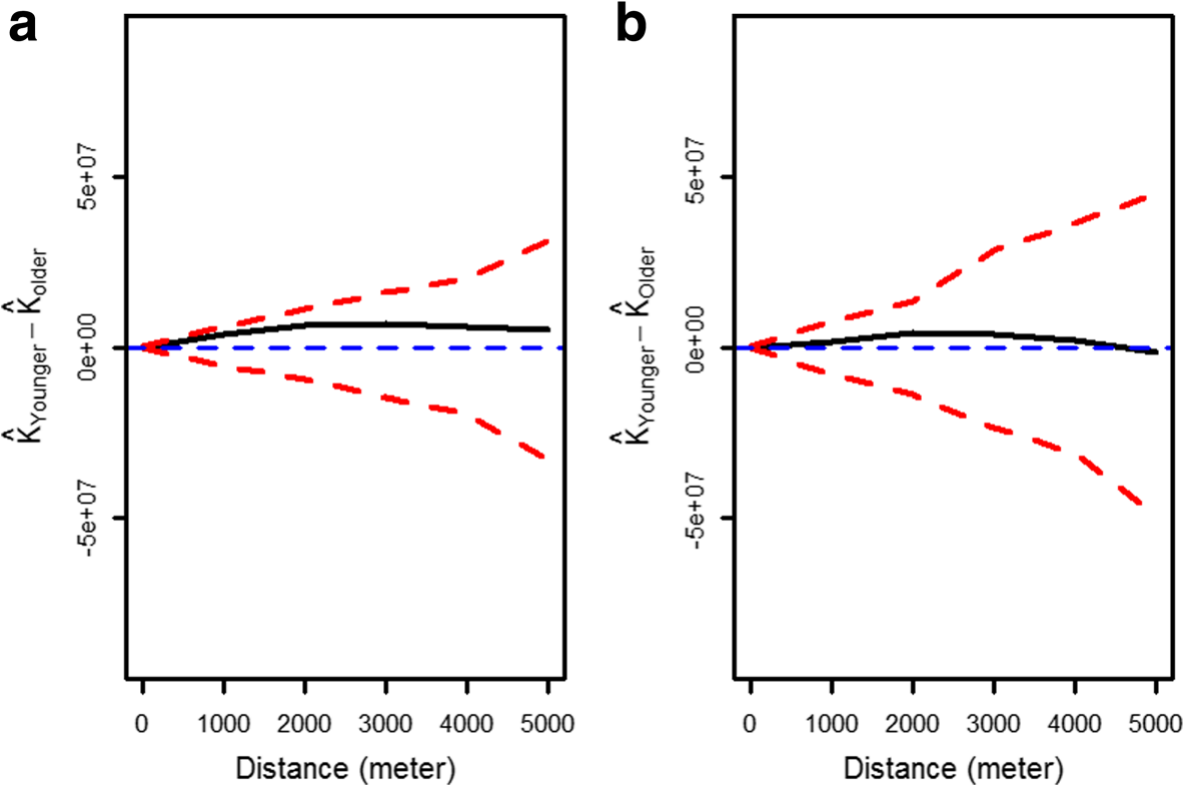
Bivariate Ripley’s K function analysis comparing the spatial distribution of two children groups who presented malaria cases, younger and older than 3 years old: **a** *P. falciparum* incidence during the two-year follow-up, **b** *P. vivax* incidence during the two-year follow-up. The *blue line* represents the expected difference of K function values (K_infected_-K_noninfected_) between children groups under the null hypothesis of spatial independence, the *solid black line* represents the observed difference K function, *dashed red lines* represents the confidence envelopes for expected K-function values calculated from 999 simulations

Univariate K-function values for both species indicated that children with episodes were significantly clustered at all distances up to 5.0 km in both years of follow-up (Additional file 5). According to the bivariate K-function plots, the distribution of children with *P. falciparum* episodes in the first year were significantly more clustered than children without episodes only at distances greater than 4.0 km (Fig. 2a); while in the second year, this comparative increased clustering pattern of children with episodes occurred at all distances (Fig. 2b). Regarding *P. vivax*, children with episodes in the first year were significantly more clustered than those without episodes at distances lower than 1.5 km, and those larger than 3.5 km (Fig. 2c). In the second year, children with *P. vivax* episodes were significantly more clustered only at distances larger than 4.2 km (Fig. 2d). On the other hand, K-function analyses also indicated that children younger than 3 years and those older than 3 years, both with malaria episodes, were independently spatial distributed (Fig. 3).

### Local spatial clustering

**Fig. 4:**
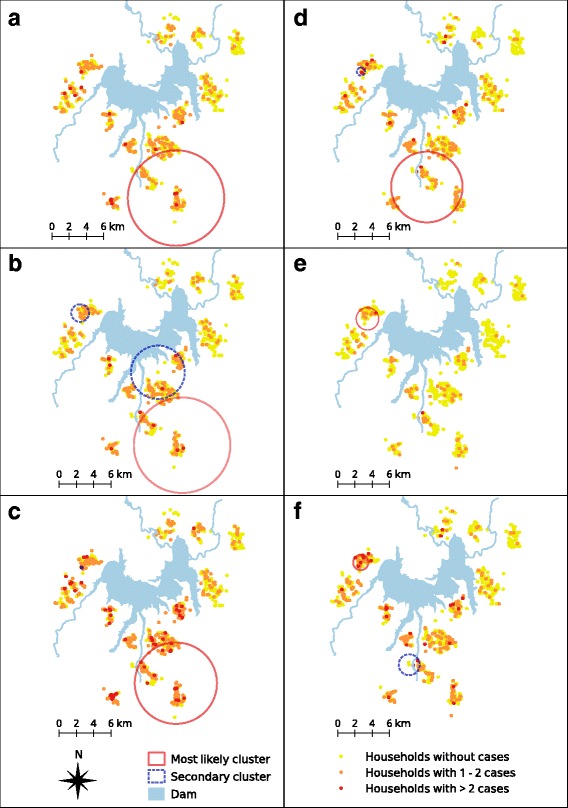
The most likely and secondary spatial clusters of *P. falciparum* and *P. vivax* incidence. *P. falciparum*: **a** (first year), **b** (second year) and **c** (both years); *P. vivax:* **d** (first year), **e** (second year), **f** (both years)

Purely spatial analysis by SaTScan confirmed that malaria episodes due to both species were not randomly distributed. The most likely spatial cluster of *P. falciparum* incidence in year 1 was a 5.5 km radius area south of the dam, composed of 167 HH presenting a total 81 episodes (Fig. 4a; Table 2). Households within this cluster belonged mainly to Kara and Yasso villages and were 1.8 times more at risk of acquiring *P. falciparum* infections than households outside the cluster (RR = 1.8, *P* = 0.02). In year 2, the most likely cluster of *P. falciparum* episodes was also located south of the dam with a radius area of 3.1 km, including 133H of mainly Kara and Yasso villages and accounting for 67 episodes (RR = 2.2, *P* < 0.001) (Fig. 4b; Table 2). Interestingly, the re-analysis of the children within the most likely clusters for *P. falciparum* did not identify a further hotspot in both years. In addition, two secondary clusters were identified at south and west of the dam only in the second year (Fig. 4b; Additional file 6). Both the purely spatial (Fig. 4c) and the spatio-temporal analysis (Table 3) during the two-year period consistently confirmed the location of the most likely cluster of *P. falciparum* incidence south of the dam, with the latter analysis identifying 11 weeks with the highest incidence in year 1 (September 14th - November 29th 2008) and 5 weeks in year 2 (October 11th - November 14th 2009).

**Table 2:** Spatial scan statistics of the most likely cluster of *P. falciparum* and *P. vivax* malaria episodes

	*P. falciparum*			*P. vivax*		
	Year 1	Year 2	Totals	Year 1	Year 2	Total
Coordinates (N, E)	7.6920 N, 37.3129E	7.6898 N, 37.3201E	7.7024 N, 37.3128E	7.7034 N, 37.2825E	7.8225 N, 37.2188E	7.8266 N, 37.2171E
Radius (km)	5.53	5.54	4.72	4.10	1.29	0.87
Households (%)	14.5% (167/1148)	11.6% (133/1148)	16.4% (189/1148)	11.1% (128/1148)	7.4% (85/1148)	6.01% (69/1148)
Population (%)	13.8% (282/2040)	10.5% (214/2040)	15.0% (306/2040)	11.6% (236/2040)	4.7% (96/2040)	3.8% (77/2040)
Cases	22.3% (81/363)	20.8% (67/322)	25.8% (177/685)	21.3% (63/296)	17.9% (16/89)	10.1% (39/385)
LLR	9.57	14.72	22.47	11.31	10.52	14.56
Relative risk	1.79	2.24	1.87	2.07	4.44	2.83
*P*-value	0.02	< 0.001	< 0.001	0.004	0.008	< 0.001

*Abbreviation*: *LLR* log likelihood ratio

**Table 3:** Space-time scan statistics of the most likely cluster of *P. falciparum* and *P. vivax* malaria episodes

	*P. falciparum*		*P. vivax*	
	Year 1	Year 2	Year 1	Year 2
Time period (initial - final)	14/09/08–29/11/08	11/10/09–14/11/09	17/08/08–29/11/08	15/11/09–13/02/10
Coordinates (N, E)	7.6806N, 37.2524E	7.7391N, 37.3161E	7.7002N, 37.3172E	7.7243N, 37.2763E
Radius (km)	6.83	4.09	5.22	2.19
Households (%)	12.6% (145/1148)	16.2% (186/1148)	16.9% (194/1148)	4.9% (56/1148)
Population (%)	14.4% (295/2040)	14.5% (297/2040)	14.8% (302/2040)	5.11% (104/2040)
Cases	9.3% (34/363)	15.2% (49/322)	15.9% (47/296)	12.3% (11/89)
LLR	20.97	73.70	29.57	15.09
Relative risk	4.03	11.90	4.22	10.24
*P*-value	< 0.001	< 0.001	< 0.001	0.012

*Abbreviation*: *LLR* log likelihood ratio

The most likely spatial clusters of *P. vivax* incidence were a 4.1 km radius area located south of the dam in year 1 (Fig. 4d; Table 2) and a 1.3 km radius area west of the dam in year 2 (Fig. 4e; Table 2). The first cluster included 128 HHs of mainly Yasso village and presenting 63 episodes (RR = 2.1, *P* = 0.004) while the second one included 88 HHs in Buddo village presenting 16 episodes (RR = 4.4, *P* = 0.008). The re-analysis of the children within the most likely clusters for *P. vivax* did not identify a further hotspot in both years. In addition, two small secondary clusters were identified west of the dam only in year 1; one of them had a similar location to the most likely cluster identified in the following year (year 2) (Fig. 4d; Additional file 6). The spatio-temporal analysis confirmed the most likely clusters south and west of the dam respectively in year 1 and year 2. Fifteen weeks with highest *P. vivax* incidence were identified in year 1 (August 17th - November 29th 2008), and 13 weeks in year 2 (November 15th 2009 - February 13th 2010).

## Discussion

Species-specific malaria episodes in children under 10 years old detected by ACD between July 2008 and June 2010 were clustered in groups of HHs (hotspots) in selected villages around the Gilgel-Gibe hydropower dam in Southwest Ethiopia. Like in other endemic Ethiopian regions [9, 26], *P. falciparum* episodes predominated over *P. vivax* episodes with *P. vivax* showing a sharp decrease in annual incidence rates during the study period. Comparatively, hotspots of *P. falciparum* incidence in children were more stable at a geographical level and over time than those of *P. vivax* incidence, with consistent locations at the south of the dam in the two successive study years.

The level of statistical significance is an important factor in determining whether a certain geographical area forms a plausible hotspot of malaria transmission. In this study, the global clustering K-function test suggested the existence of clustering of children with malaria episodes without pinpointing specific locations, while its variant, the bivariate K-function, was able to demonstrate that children with malaria episodes tended to be more aggregated than children without episodes. Although a previous study using recurrent-event models to analyse incidence data in the same study children showed contrasting associations between the age of children and the species-specific malaria incidence (i.e. *P. vivax* were mostly observed in younger age groups, while *P. falciparum* episodes were mainly seen in older children) [18], the age would not have any influence over the spatial distribution of children presenting species-specific malaria episodes according to the bivariate K-function analysis.

In contrast to global clustering tests, local clustering tests (i.e. the Kulldorf spatial scan statistic) were able to identify the most likely location of hotspots of malaria incidence in the two consecutive years of study. Indeed, hotspots of *P. falciparum* incidence suggested a higher exposure to infectious mosquitoes in southern villages, especially after the long rainy season (peak of cases between September and November according to space-time analysis). After rains, intermittent streams could create pockets or pools of water which can serve as potential breeding sites for mosquitoes *Anopheles arabiensis*, contributing to an increase in mosquito density and vector-human contacts, and consequently to a greater number of malaria episodes during the dry season [18, 27]. The characteristics of the southern land (i.e. wet, flat and silted) [28], as well as, the landslides that often occur there [29, 30], would additionally increase the accumulation of water in shallow pits that act as excellent mosquito breeding habitats. Of note, as previously found in a recent article [18], the Gilgel-Gibe dam reservoir would not have a significant impact on the malaria transmission in the study area, since the design and automatic operation of the dam would be able to prevent the appearance of shoreline puddles and consequently the formation of mosquito breeding sites near the reservoir.

In contrast to *P. falciparum*, the hotspots of *P. vivax* incidence were less stable in place and time during the study period, suggesting that the occurrence of *P. vivax* clinical episodes are less sensitive to seasonal and environmental changes than *P. falciparum*, and that other factors should be considered to understand the spatial-temporal heterogeneity of infections due to this species [18]. The biological features of *P. vivax* infections may also influence the spatial distribution of the disease, particularly the ability of parasites to relapse weeks or months after a primary parasitaemia [31]. However, the characterization and prediction of spatial patterns remain challenging because of the difficulty distinguishing between a hypnozoite-triggered relapse, a resurgence of erythrocytic parasites (i.e. recrudescence) due to a failure in the therapy, or reinfection of an individual with a new parasite strain following a primary infection [32, 33]. This challenge is even greater considering that children with confirmed *P. vivax* episodes in the study received chloroquine (CQ) but not primaquine (PQ), following the national guidelines for areas where the glucose-6-phosphate dehydrogenase deficiency (G6PD deficiency) is not known, and where tests to detect that condition are not available [19].

The immunity to the malaria infection is another factor that should be considered when interpreting the spatial and spatial-time patterns of malaria clinical episodes in study children. As previously hypothesised in a recent article [18], differences in clinical malaria incidence between species with respect to age may be related to different species-specific acquisition rates of immunity [34], with immunity acquired more rapidly with *P. vivax* than with *P. falciparum*. Taking this into account, the fast development of clinical immunity in areas with the highest *P. vivax* exposure and incidence (i.e. southern villages) identified during the first study year may also explain why the hotspots did not remain in the same location in the following year. Similarly, immunity would be a factor to be considered in the analysis of the reduction of *P. vivax* clinical incidence rates during the study period [18].

The main limitations of our study may be related to the malaria metrics and the geospatial analysis used for the identification of clusters of malaria transmission in the area. The spatial analysis of clinical malaria incidence obtained through rigorous weekly active case detection of symptomatic episodes in enrolled children may be the best method for detecting malaria hotspots if most malaria infections occurred in those children were symptomatic and microscopically detected [6]. However, this cannot be confirmed in this study because the methodology did not include the screening for asymptomatic and sub-microscopic malaria infections. Malaria surveys in other endemic regions of Ethiopia have reported that asymptomatic and sub-microscopic infections can represent an important proportion of total malaria infections [35, 36]; however, the cross-sectional design in the latter studies did not take into account the incubation period for some of those infections, hence the potential development of symptoms and the increase of parasite density levels at a later stage [37], which would facilitate their detection by surveillance methods with strict follow-up like those included in our study. Further research is needed to better understand the impact of asymptomatic and sub-microscopic infections in endemic areas of Ethiopia, and to assess whether their spatial distribution differs from that of symptomatic infections. On the other hand, despite the recognition of SaTScan as a powerful tool to analyse spatial patterns of vector-borne diseases such as malaria [38, 39], a number of studies have pointed out that setting critical parameters in the analysis such as the maximum window size is not straightforward and suggested that this task should consider the application goals of the cluster detection and geographic scale of processes leading to the clusters [40]. Following these suggestions, our analysis set the maximum window size at 15%, instead of the default value of 50%, with the purpose of detecting the zones with the highest malaria incidence within the entire study area. This chosen parameter value did not only avoid large non-populated areas within circular clusters but also reduced (without eliminating) the influence of the uneven inter- and intra-village distribution of children/households in the study area. Moreover, the no identification of smaller and more homogeneous clusters with even higher transmission among the most likely malaria clusters of children/households (i.e. no more hotspots within hotspots), further supports our selection for the maximum window size, as well as the validity of the, detected most likely clusters for *P. falciparum* and *P. vivax* incidence (despite being composed of most households of a village or a group of contiguous villages). Other limitations of the study may be related to the absence of data on other potential risk factors for malaria infection (e.g. household size, education level in parents, malaria prevention practices at household, treatment-seeking behaviour, vegetation coverage, etc.) preventing the evaluation of their influence on the spatial distribution of the clinical malaria incidence. Gender and distance to the Gilgel-Gibe dam were not considered as covariates for the spatial analysis since those variables were not significantly associated with both *P. vivax* and *P. falciparum* malaria incidence in a longitudinal modelling approach [18]; while the children’s age (despite being associated with malaria incidence according to the same model), which was found to be evenly distributed across villages, may not have influence over the spatial distribution of children with malaria episodes in the study area according to the K-function test, hence not meeting the criteria to be catalogued as covariate [24].

## Conclusion

Hotspots of *P. falciparum* incidence in children were more stable at the geographical level and over time than *P. vivax* incidence in selected villages around the Gilgel-Gibe hydropower dam in Southwest Ethiopia. Different malaria spatial-time patterns due to *Plasmodium falciparum* and *Plasmodium vivax* should be taken into account to better design and deliver targeted interventions.

## Additional files

Additional file 1: Selection of window size for SaTScan analysis. (DOCX 17 kb)

Additional file 2: Spatial scan statistics of the most likely cluster of malaria episodes using different maximum window sizes. (DOCX 16 kb)

Additional file 5: Ripley’s K function analyses showing significant spatial clustering of children with malaria episodes. *P. falciparum*: a (first year), b (second year); *P. vivax*: c (first year), d (second year). The red line represents the expected K function values under the null hypothesis of complete spatial randomness, the solid black line represents observed K function values, and the grey area represents confidence envelopes for expected K-function values calculated from 999 simulations. (TIF 7910 kb)

Additional file 6: Spatial scan statistics of the secondary clusters of *P. falciparum* and *P. vivax* malaria episodes by year of study. (DOCX 15 kb)

## Abbreviations

ACD: Active case detection
AL: Artemether-lumefantrine
CE: Confidence envelopes
CI: Confidence interval
CQ: Chloroquine
CSR: Complete spatial randomness
EFY 2014/2015: Ethiopian fiscal year 2014/2015
GPS: Global positioning system
HH: Household
LLR: Log likelihood ratio
LM: Light microscopy
PCD: Passive case detection
RDT: Rapid diagnostic test
RR: Relative risk
