# Supplementary material for: Household level spatio-temporal analysis of Plasmodium falciparum and Plasmodium vivax malaria in Ethiopia
Source: Parasit Vectors. 2017 Apr 20;10:196. doi: 10.1186/s13071-017-2124-6 (PMC5397782; doi:10.1186/s13071-017-2124-6)
Supplement: Supplementary file 3 — Selection of window size for SaTScan analysis. (DOCX 17 kb) [file 13071_2017_2124_MOESM1_ESM.docx]

**ADDITIONAL FILE 1**

The maximum spatial cluster size was set at 15% of the population at risk (children), after evaluating the effects of switching this parameter on the identification of clusters by SaTScan.

The default parameter of 50% identified that the most likely *P. falciparum* cluster had a radius of 8.2 km and that the most likely *P. vivax* cluster had a radius of 7.8 km (See Additional file 2). However, in both cases the cluster circle included a large study area without households where children lived, as well as an important extension of the dam. A maximum spatial cluster size of 30% allowed for the identification of a most likely *P. falciparum* spatial cluster with a radius of 7.73 km and a *P. vivax* cluster with a radius of 7.17 km. Again, the cluster circle included a large study area without households, and an important proportion of the dam. The maximum spatial cluster size of 20% reduced slightly the cluster radius for *P. falciparum* (4.77 km), but no changes were found for *P. vivax* cluster (7.17 km) resulting in again large areas within the cluster circle without households, especially for *P. vivax*. Finally, when the maximum spatial cluster size was set in 15%, SaTScan analysis identified a most likely cluster of 4.47 Km radius for *P. falciparum* cluster and a cluster of 0.87 Km radius for *P. vivax*, and the cluster circle did not include any area of dam, and the area without households was reduced for both species.
