# Supplementary material for: Household level spatio-temporal analysis of Plasmodium falciparum and Plasmodium vivax malaria in Ethiopia
Source: Parasit Vectors. 2017 Apr 20;10:196. doi: 10.1186/s13071-017-2124-6 (PMC5397782; doi:10.1186/s13071-017-2124-6)
Supplement: Supplementary file 4 — Spatial scan statistics of the most likely cluster of malaria episodes using different maximum window sizes. (DOCX 16 kb) [file 13071_2017_2124_MOESM2_ESM.docx]

**ADDITIONAL FILE 2**

**Table S1. Spatial scan statistics of the most likely cluster of *P. falciparum* malaria episodes using different maximum windows size**

|  | **50%** | **30%** | **20%** | **15%** |
| --- | --- | --- | --- | --- |
| Coordinates (N, E) | 7.7472 N,  37.2822 E | 7.6873 N,  37.3145 E | 7.70196 N,  37.2864 E | 7.7024 N,  37.3128 E |
| Radius (Kilometres) | 8.27 | 7.73 | 4.77 | 4.72 |
| Households (%) | 45.6%  (524/1148) | 28.8%  (331/1148) | 19.1%  (219 / 1148) | 16.4%  (189/1148) |
| Population (%) | 44.6%  (910/2040) | 28.5%  (582/2040) | 20.0%  (407/2040) | 15.0%  (306/2040) |
| Cases | 67.7%  (464/685) | 44.4%  (304/685) | 32.3%  (221/685) | 25.8%  (177/685) |
| LLR | 47.86 | 38.30 | 28.84 | 22.47 |
| Relative risk | 2.16 | 2.01 | 1.91 | 1.87 |
| P-value | <0.001 | <0.001 | < 0.001 | < 0.001 |

**LLR: Log Likelihood Ratio**

**Table S2. Spatial scan statistics of the most likely cluster of *P. vivax* malaria episodes using different maximum windows size**

|  | **50%** | **30%** | **20%** | **15%** |
| --- | --- | --- | --- | --- |
| Coordinates (N, E) | 7.7356 N,  37.2994 E | 7.685549 N, 37.316017 E | 7.685549 N, 37.316017 E | 7.826615 N, 37.217100 E |
| Radius (Kilometres) | 7.89 | 7.17 | 7.17 | 0.87 |
| Households (%) | 42.5%  (488/1148) | 20.9%  (239/1148) | 20.8  (239/1148) | 6.01%  (69/1148) |
| Population (%) | 44.6%  (910/2040) | 19.2%  (392/2040) | 19.2%  (392/2040) | 3.8%  (77/2040) |
| Cases | 63.1%  (243/385) | 32.7%  (126/385) | 32.7%  (126/385) | 10.1  (39/385) |
| LLR | 26.41 | 19.21 | 19.21 | 14.56 |
| Relative risk | 2.12 | 2.04 | 2.04 | 2.83 |
| P-value | < 0.001 | < 0.001 | < 0.001 | < 0.001 |

**LLR: Log Likelihood Ratio**
