# Supplementary figures and images for: Household level spatio-temporal analysis of Plasmodium falciparum and Plasmodium vivax malaria in Ethiopia
Source: Parasit Vectors. 2017 Apr 20;10:196. doi: 10.1186/s13071-017-2124-6 (PMC5397782; doi:10.1186/s13071-017-2124-6)

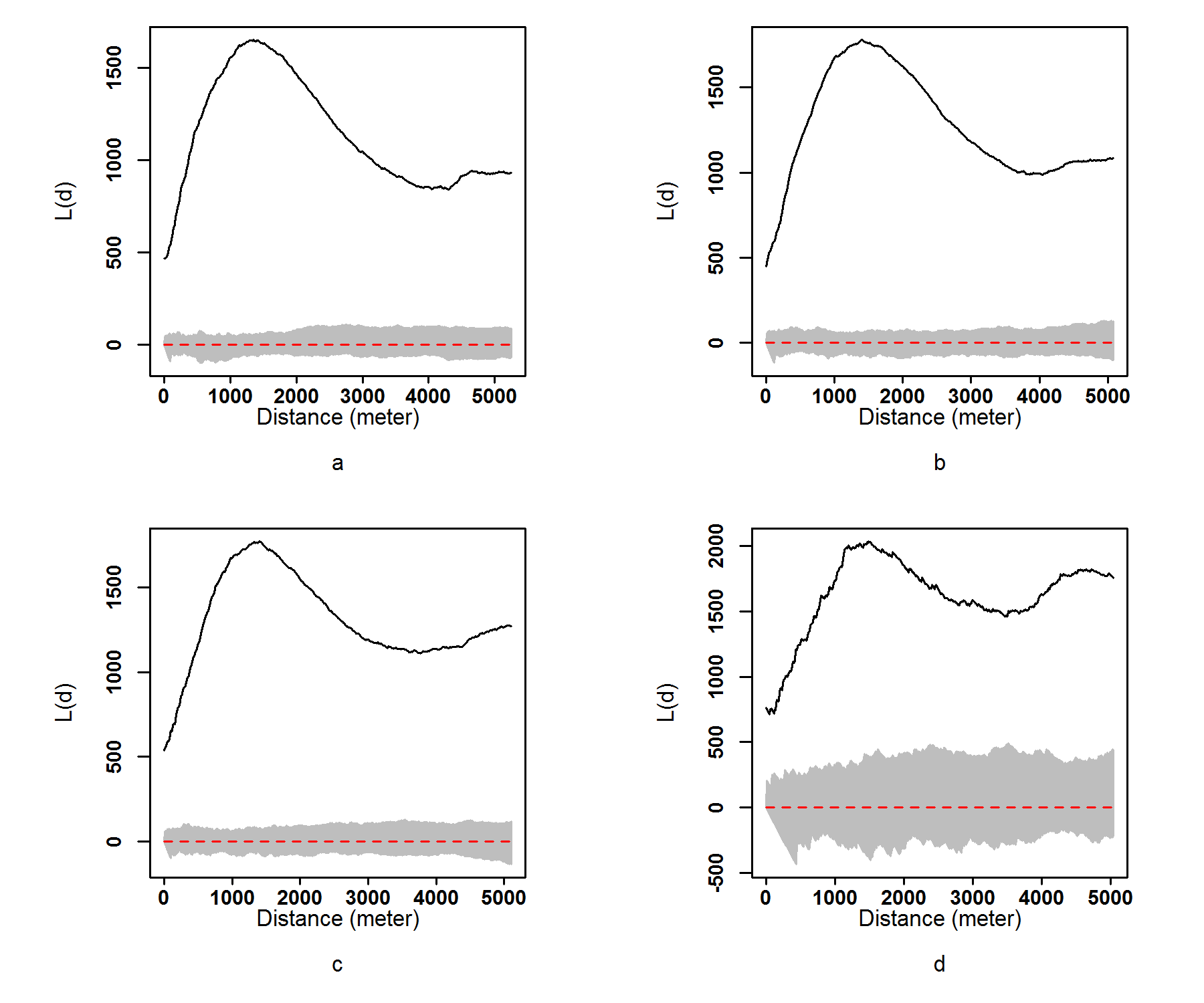

Supplement: Supplementary file 5 — Ripley’s K function analyses showing significant spatial clustering of children with malaria episodes. P. falciparum: a (first year), b (second year); P. vivax: c (first year), d (second year). The red line represents the expected K function values under the null hypothesis of complete spatial randomness, the solid black line represents observed K function values, and the grey area represents confidence envelopes for expected K-function values calculated from 999 simulations. (TIF 7910 kb) [file 13071_2017_2124_MOESM5_ESM.tif]
