# Supplementary material for: Household level spatio-temporal analysis of Plasmodium falciparum and Plasmodium vivax malaria in Ethiopia
Source: Parasit Vectors. 2017 Apr 20;10:196. doi: 10.1186/s13071-017-2124-6 (PMC5397782; doi:10.1186/s13071-017-2124-6)
Supplement: Supplementary file 6 — Spatial scan statistics of the secondary clusters of P. falciparum and P. vivax malaria episodes by year of study. (DOCX 15 kb) [file 13071_2017_2124_MOESM6_ESM.docx]

**Additional file 6. Spatial scan statistics of the secondary clusters of *P. falciparum* and *P. vivax* malaria episodes by year of study**

|  | ***P. falciparum*** | | ***P. vivax*** | | |  |
| --- | --- | --- | --- | --- | --- | --- |
|  | ***Year 2*** | | ***Total*** | ***Year 1*** | | ***Total*** |
| Coordinates (N, E) | 7.8276 N, 37.2139 E | 7.7658 N, 37.2949 E | 7.8228 N, 37.2147 E | 7.8238 N, 37.2138 E | 7.83134N, 37.2202 E | 7.7200 N, 37.2676 E |
| Radius (Kilometres) | 1.02 | 3.09 | 0.098 | 0.47 | 0.00 | 1.21 |
| Households (%) | 6.2% (72/1148) | 14.4% (166/1148) | 0.26% (3/1148) | 1.3% (15/1148) | 0.08% (1/1148) | 1.3% (16/1148) |
| Population (%) | 4.0% (82/2040) | 14.2% (290/2040) | 0.15% (3/2040) | 0.8% (16/2040) | 0.09% (2/2040) | 2.1% (43/2040) |
| Cases | 10.2% (33/322) | 23.3% (75/322) | 1.30% (9/685) | 4.1% (12/296) | 1.4%  (4/296) | 7.0% (27/385) |
| LLR | 11.54 | 9.44 | 22.47 | 10.18 | 9.28 | 14.01 |
| Relative risk | 2.73 | 1.83 | 1.87 | 5.31 | 25.92 | 3.49 |
| P-value | 0.003 | 0.025 | 0.003 | 0.012 | 0.038 | <0.001 |

**LLR: Log Likelihood Ratio**
